# Psychrotrophic bacteria in milk: How much do we really know?

**DOI:** 10.1590/S1517-838246220130963

**Published:** 2015-06-01

**Authors:** Gislene B. de Oliveira, Luciana Favarin, Rosa H. Luchese, Douglas McIntosh

**Affiliations:** 1 Universidade Federal Rural do Rio de Janeiro, Ciência e Tecnologia de Alimentos, Universidade Federal Rural do Rio de Janeiro, Seropédica, RJ, Brasil, Ciência e Tecnologia de Alimentos, Universidade Federal Rural do Rio de Janeiro, Seropédica, RJ, Brazil.; 2 Universidade Federal Rural do Rio de Janeiro, Parasitologia Veterinária, Universidade Federal Rural do Rio de Janeiro, Seropédica, RJ, Brasil, Parasitologia Veterinária, Universidade Federal Rural do Rio de Janeiro, Seropédica, RJ, Brazil.

**Keywords:** microbiology, *Pseudomonas*, dairy products, microbial identification

## Abstract

The occurrence of psychrotrophic bacteria in raw milk is studied worldwide due to the difficulties associated with controlling their growth during cold storage and the consequent negative effects upon fluid milk or dairy products. Among the psychrotrophic bacteria, the genus *Pseudomonas* (represented primarily by *P. fluorescens*) has been highlighted as the cause of numerous defects in dairy products. In light of its perceived predominance, this species has frequently been chosen as a model organism to assess the effects of psychrotrophic bacteria on milk or to evaluate the efficacy of control measures. However, recent findings derived from the application of molecular biological techniques have exposed a number of deficiencies in our knowledge of the biology of milk-associated psychrotrophs. Furthermore, it has been revealed that microbe to microbe communication plays a significant role in determining both the identities and the extent to which different groups of microbes develop during cold storage. The application of molecular identification methods has exposed errors in the classification of members of the genus *Pseudomonas* isolated from cold stored milk and has stimulated a reevaluation of the presumed status of *P. fluorescens* as the predominant milk-associated psychrotrophic species. This article presents a succinct review of data from studies on psychrotrophic bacteria in milk, some of which contest established theories in relation to the microbiology of cold stored raw milk, and poses the question: how much do we really know?

## Introduction

The term psychrotrophs (also denominated psychrotolerant) refers to microorganisms that have the ability to grow at low temperatures but have optimal and maximal growth temperatures above 15 and 20 °C, respectively (Moyer and Morita, 2007). This characteristic makes these microbes especially significant with regard to food spoilage and safety, given that the storage of many foods at cold temperatures is a routine practice during production, transportation, processing and post-purchase (Beales, 2004; Russell, 2002).

Raw milk provides a physicochemical environment that is favourable for the multiplication of a broad spectrum of microorganisms, including a range of psychrotrophic bacterial species (predominantly members of the genus *Pseudomonas*) that contaminate milk during collection and/or processing (Mcphee and Griffiths, 2011; Pinto *et al.*, 2006; Sørhaug and Stepaniak, 1997).

Although the pasteurisation of raw milk decreases its microbial load, the efficiency of the process and the resulting quality of the dairy products are directly influenced by the microbiological quality of the raw milk (Nörnberg *et al.*, 2010). Rigorous hygiene standards employed to reduce the possibility of exogenous contamination coupled with low storage temperatures to control the growth of mesophilic organisms are essential components of the microbial control strategies employed with raw fluid milk (Barbano *et al.*, 2006). Approaches including enclosed pipeline milk systems, better sanitary design of equipment, cleaner cows, and more effective "cleaning in place" coupled with the rapid cooling of raw milk using in-line plate coolers prior to storage in bulk tanks have been shown to reduce the growth of contaminating bacteria (Barbano *et al.*, 2006).

The presence and subsequent replication of populations of psychrotrophs may lead to the spoilage of milk (Beales, 2004; Nörnberg *et al.*, 2010; Pinto *et al.*, 2006). Because the economic impact of this group of microbes upon the global dairy industry is substantial, psychrotrophic bacteria have been and continue to be studied extensively with the main objectives of developing effective control measures and establishing regulations to ensure the quality and safety of milk and dairy products (Mcphee and Griffiths, 2011).

Despite the existence of an extensive body of published data, based on the results of recent studies it would be fair to state that numerous gaps exist in our understanding of the biology of the psychrotrophic bacteria of importance for the dairy industry. The continued development of molecular tools for bacterial identification and their application to the analysis of microbial population structures and ecology in milk and dairy products has revealed the presence of psychrotrophic bacteria undetected by the use of traditional culture-based approaches (Almeida and Araujo, 2013; Marchand *et al.*, 2009b; Raats *et al.*, 2011). Similarly, molecular methods have highlighted discrepancies in the identification of the psychrotrophic species associated with the spoilage of cold stored milk, particularly with regard to the taxonomically complex genus *Pseudomonas* (Mcphee and Griffiths, 2011).

The majority of studies on milk-associated psychrotrophs have focused on individual isolates grown as planktonic cultures (readily culturable). However, there is an increasing recognition that such approaches overlook potential interactions and cross-talk between different species of psychrotrophic bacteria, many of which are non-culturable, that are present within the biofilms that develop in milk storage and processing environments and that may exert an influence on milk quality and safety (Cleto *et al.*, 2012; Marchand *et al.*, 2012).

This current mini-review sought to collate and evaluate the findings of recent studies on psychrotrophic bacteria of importance to the dairy industry and to demonstrate that the activities of psychrotrophic bacteria in milk are more extensive and more complex than was previously thought.

## Biochemical Basis for Psychrotrophic Growth

Temperatures influence bacterial growth rates by affecting the conformation of cellular macromolecules and other constituents, thereby determining the rates of intracellular enzymatic reactions that are crucial for viability (Beales, 2004; Fonseca *et al.*, 2011; Russell, 2002). Hence, the adaptation of the cell to low temperatures requires enzymes that are active in this condition (Chattopadhyay, 2006).

Under low temperature growth conditions psychrotrophic bacteria synthesise phospholipids and neutral lipids containing increased proportions of unsaturated fatty acids, resulting in a reduction in the melting point of the lipids. This phenomenon serves to maintain their fluidity, thus allowing the continued functionality, solute transport, secretion of extracellular enzymes and fluidity of the membrane (Beales, 2004; Jay, 2005)

Indeed, transcriptomic analysis of *Pseudomonas putida* strain KT2440 revealed that the expression of at least 266 genes (nearly 5% of the genome) was modified during low temperature growth in comparison to cells grown at 30 °C. Several of these changes seemed to be directed towards neutralising problems created by low temperatures, such as increased protein misfolding, increased stability of DNA/RNA secondary structures, reduced membrane fluidity and reduced growth rates (Fonseca *et al.*, 2011).

## Psychrotrophic Bacteria in Milk

Refrigeration alone or in combination with other methods such as the addition of preservatives is the most commonly used means of preserving food, including milk and dairy products (Beales, 2004). The current trend in the dairy industries is to reduce the frequency of milk collection; thus, the refrigerated storage of milk has been lengthened from two to five days prior to heat treatment (O'brien and Guinee, 2011). This practice has been stimulated in part by a desire for a 5-day work week and in response to a decreased milk supply at certain times of the year (Mcphee and Griffiths, 2011).

The procedure of cooling and the subsequent refrigerated storage of raw milk effectively controls the development of populations of mesophilic spoilage organisms while at the same time providing a selective advantage for the growth of psychrotrophic bacteria (Barbano *et al.*, 2006; De Jonghe *et al.*, 2011; Samarzija *et al.*, 2012). According to Sørhaug and Stepaniak (1997) and Mcphee and Griffiths (2011), the cultivable psychrotrophic bacteria in milk are represented predominantly by Gram-negative genera including *Pseudomonas*, *Achromobacter*, *Aeromonas*, *Serratia*, *Alcaligenes*, *Chromobacterium* and *Flavobacterium* spp., and at much lower numbers by Gram-positive genera including *Bacillus*, *Clostridium*, *Corynebacterium*, *Streptococcus, Lactobacillus* and *Microbacterium* spp.

Interestingly, milk freshly drawn from the udder often does not contain detectable populations of culturable psychrotrophic bacteria. However, these populations develop over time in virtually all cold stored raw milk, a feature that reduces the normal refrigerated storage life to less than 5 d (Chen *et al.*, 2003; Ma *et al.*, 2003; Mcphee and Griffiths, 2011; Raats *et al.*, 2011). The average counts of psychrotrophic aerobic bacteria in milk silos at several dairies in southwest Scotland were reported to be 1.3 × 10^5^ cfu mL^−1^. The majority of the bacteria present were Pseudomonads (70.2%), but Enterobacteriaceae (7.7%), Gram-positive bacteria (6.9%), and other Gram-negative, rod-shaped organisms were also isolated (Mcphee and Griffiths, 2011). Following storage for a further 48 h at 6 °C, the psychrotrophic counts increased by two log cycles to 1.3 × 10^7^ cfu mL^−1^.

In addition to growing at low temperatures, a variety of psychrotrophic bacterial species (primarily represented by pseudomonads) found in raw milk produce heat-stable proteases (Liu *et al.*, 2007) and lipases (Chen *et al.*, 2003), generally during the late log or early stationary growth phases when the cell density is high. Many of the produced enzymes retain significant activity after pasteurisation (72–75 °C/15–20 s) and even UHT treatment (130–150 °C/2–4 s), and may subsequently degrade proteins and fats present in the processed products (Barbano *et al.*, 2006; De Jonghe *et al.*, 2011; Dunstall *et al.*, 2005).

A reduction in cheese yield and tainting are the two most frequently reported negative effects in cheese production that are attributed to psychrotrophic-derived enzymes (Mcphee and Griffiths, 2011). Less frequently reported effects include the alteration of starter activity and/or growth rates and rennet coagulation times (Datta and Deeth, 2001; Mankai *et al.*, 2012). Reduced yields in cheese production occur mainly because soluble casein degradation products (peptides and amino acids) may be lost into the whey instead of forming part of the curd (Mcphee and Griffiths, 2011). The tainting problems are due to the action of proteases, which generate bitter flavours, and lipases, which hydrolyse milk fat yielding free fatty acids (FFAS) and generate strong flavours that in the majority of cases are considered undesirable (Deeth, 2006; Mankai *et al.*, 2012).

‘Age gelation' of UHT milk is an irreversible phenomenon characterised by a change in the physical state that is manifested by a rise in viscosity of more than 10 mPa.S (at 20 °C), followed by the formation of a gel and loss of fluidity (Datta and Deeth, 2001). According to Sørhaug and Stepaniak (1997), a psychrotrophic population of 5.5 log cfu mL^−1^ in raw milk causes UHT milk gelation after 20 weeks of storage, while populations between 6.9 and 7.2 logs will cause the same effect between 2 and 10 weeks.

## Biofilm Formation

The procedures of cooling and refrigeration of milk are not guarantees of quality. The first point of control is to ensure that raw milk is obtained under sanitary conditions designed to minimise contamination (Beales, 2004). The second point of control is dependent on the adequate cleaning and disinfection of all of the equipment used for the collection, transport, and storage of refrigerated raw milk. This step is necessary to prevent fouling with milk film, which can support the growth of bacteria as multi-species biofilms that represent a source of contamination for any subsequent batches of milk (Mcphee and Griffiths, 2011; Perin *et al.*, 2012).

Biofilms are surface-associated bacterial communities that are embedded in a matrix of self-produced polymeric substances (EPSs) consisting of nucleic acids, polysaccharides, lipids and proteins resulting from the successful attachment and subsequent growth of microorganisms on a surface (Marchand *et al.*, 2012; Toyofuku *et al.*, 2012). In nature biofilms can be composed of a single species, but more commonly they comprise a consortium of species (Skandamis and Nychas, 2012). The critical stages for biofilm development are adherence, proliferation, and the dispersion phases (Li and Tian, 2012). Each of these stages includes reinforcement by or modulation of the extracellular matrix. However, the functionality of biofilms depends on a complex web of symbiotic interactions and factors, including pH, nutrient availability, quorum sensing molecules, the presence of organic and inorganic compounds and temperature (Bai and Rai, 2011; Oliveira *et al.*, 2010). Biofilms are the predominant form of growth for bacteria in the majority of environments, including food processing settings (Bai and Rai, 2011; Cleto *et al.*, 2012). More recently, the production of spoiling enzymes by bacteria present in biofilms has also been studied (Teh *et al.*, 2014).

Many *Pseudomonas* species utilise biofilm formation during plant colonisation to enhance persistence, resulting in the production of a variety of biofilm matrix molecules (Mann and Wozniak, 2012). An intriguing feature of milk-spoiling *Pseudomonas* recovered from biofilms is their ability to alter phenotypes via the process of phase variation (Marchand *et al.*, 2012). Through this process, high-frequency phenotypic switching is mediated by mutation, reorganisation, or modification of the genome (Van Den Broek *et al.*, 2005), contributing to the survival of the biofilm population during environmental stresses such as temperature fluctuations and frequent exposure to sanitizers during the cleaning of dairy processing and storage equipment (Marchand *et al.*, 2012). The limited experimental data generated to date indicates that the traditional approach of studying milk spoilage organisms as planktonic monocultures has most likely served to distort and misdirect our understanding of spoilage processes *in situ*, and future studies would undoubtedly benefit from experimental approaches designed to mimic the biofilms found in bulk storage and processing systems.

## Quorum Sensing and Metabolic Regulation

Bacteria communicate with each other using chemical signalling molecules when specific cell densities are reached via a process termed quorum sensing (Fuqua *et al.*, 1994; Liu *et al.*, 2007). As a consequence of this process gene expression can either be activated or repressed, and the behaviour of populations of single cells are synchronised in a manner similar to multi-cellular organisms (Bai and Rai, 2011; Smith *et al.*, 2004). Cell density-dependent signalling systems in bacteria control a range of phenotypic traits, including biofilm development, bioluminescence, cell differentiation, competence for DNA uptake, pigment production, conjugal plasmid transfer, production of degradative extracellular enzymes, sporulation, toxin production and virulence gene expression (Lazdunski *et al.*, 2004; Li and Tian, 2012).

N-acyl-homoserine lactones (AHLs) are quorum sensing signalling molecules that are produced by a wide range of Gram-negative bacteria, including *P. fluorescens* (Cha *et al.*, 1998; Liu *et al.*, 2007). The production of various AHLs in both raw and pasteurised milk by psychrotrophic *Pseudomonas* spp. indicates that quorum sensing may play a role in the spoilage of milk and dairy products (Pinto *et al.*, 2007). Coincidentally, the production of extracellular proteases in *P. fluorescens* is associated with the high cell density that is typically encountered towards the end of the exponential phase of growth (Bai and Rai, 2011). The stimulation of protease production by milk-associated strains of *P. fluorescens* in response to the addition of AHL has been reported (Liu *et al.*, 2007); the authors concluded that the spoiling ability of psychrotrophic *P. fluorescens* was correlated with the ability to produce AHLs, which served to regulate the expression of extracellular proteases.

In contrast, Liu *et al.* (2006) demonstrated that AHLs did not act as regulators of proteolytic activity during the spoilage of aerobically chill-stored proteinaceous raw foods including milk. In a subsequent study, Pinto *et al.* (2010) did not detect AHL signals in the supernatants of late-exponential or stationary phase broth cultures of *P. fluorescens* strain 07A isolated from milk. The authors subsequently added synthetic AHLs or bacterial extracts containing natural AHLs to O7A cultures and found no evidence of effects upon either growth or proteolytic activity, suggesting that quorum sensing (at least via AHLs) did not regulate protease production in strain 07A.

The role of AHLs as promoters of biofilm formation in *P. fluorescens* strain B52 cultures was investigated by Allison *et al.* (1998). The addition of *N*-acyl-hexanoyl homoserine lactone to fresh growth medium enhanced biofilm production in a manner similar to that observed when supernatants from 2 day old cultures of B52 were employed as the initial growth medium. Interestingly, analysis of the spent medium using an *Agrobacter tumefaciens* indicator strain failed to detect AHLs. Nevertheless, the authors concluded that *P. fluorescens* was capable of reacting to the presence of short chain (C6) exogenous homoserines, and speculated that strain B52 produced its own signalling molecule that likely possessed a longer (> C8) fatty acyl chain that could not be detected in the *A. tumefaciens* bioassay.

Although quorum sensing signalling molecules have been detected in cold stored milk and milk derivatives, their exact role in the spoilage process is still not clear and further work on this topic is clearly warranted (Bai and Rai, 2011). Specifically, the topic of inter-species communication via broad range signalling molecules (*e.g.*, autoinducer AI-2 (Skandamis and Nychas, 2012)) has received limited attention to date, but is thought to represent a pivotal process in the development of multi-species biofilms and the coordinated expression of spoilage enzymes (Li and Tian, 2012).

## *Pseudomonas* in Milk

The genus *Pseudomonas* is the most heterogeneous and ecologically significant group of known bacteria. Owing to the fact that the nutritional requirements of *Pseudomonas* spp. are very simple, representatives of the genus have been detected in virtually all natural habitats (*e.g.*, soil, house dust, fresh water and clouds), and have also been isolated from clinical instruments, aseptic solutions, cosmetics and medical products (Franzetti and Scarpellini, 2007). As such, it is not surprising that members of the genus *Pseudomonas* have long been recognised as the predominant group of psychrotrophic bacteria recovered from spoiled refrigerated milk (Chen *et al.*, 2003). Among the pseudomonads, *P. fluorescens* is generally considered to be the principal spoilage agent of stored milk (Mcphee and Griffiths, 2011; Munsch-Alatossava and Alatossava, 2006).

The efficient cold adaption of the psychrotrophic pseudomonads is believed to be linked to the possession of elevated levels (between 59 to 72%) of unsaturated lipids in their cell membranes that impart the ability to efficiently maintain membrane functionality (specifically solute transport and the secretion of extracellular enzymes) at refrigeration temperatures (Fonseca *et al.*, 2011; Jay, 2005). Furthermore, these species are able to proliferate in milk, an environment where the concentration of free iron is low, due to the production of the diffusible fluorescent pigment pyoverdine, which acts as a siderophore, allowing the bacteria to effectively sequester iron from lactoferrin (Mcphee and Griffiths, 2011).

De Jonghe *et al.* (2011) examined the growth of psychrotrophic pseudomonads in raw milk under conditions that simulated prolonged storage (4 days on the farm, 8 hours in transport and 24 hours of storage at the dairy plant) at suboptimal (6 °C) and optimal (4 °C) storage temperatures. The numbers of *Pseudomonas* were similar during the first 72 h of storage at either temperature. However, by the end of the experiment, a striking difference of 2 log cfu mL^−1^ was reported between the optimal and suboptimal storage conditions. Moreover, *Pseudomonas* counts reached the same levels as the total aerobic plate counts by the end of the experiment (10^6^ and 10^8^ cfu mL^−1^ for optimally and sub-optimally cooled milk, respectively). Unfortunately, direct comparisons between the work of De Jonghe *et al.* (2011) and similar studies (*e.g.*, Martin *et al.*, 2011) are difficult to perform due to methodological differences and the specific strains investigated. In this context, the importance of the choice of strain was highlighted in the work of Jaspe *et al.* (1995), which demonstrated that *Pseudomonas* spp. isolated from milk that had been stored at 7 °C for three days grew ten times faster at 7 °C, had 1000-fold more proteolytic activity, and were 280-fold more lipolytic than *Pseudomonas* spp. isolated from freshly drawn milk.

Phenotypic analysis of microorganisms isolated from raw milk by Mcphee and Griffiths (2011) demonstrated that *P. fluorescens* biovar I (32.1% of isolates), *P. fragi* (29.6%), *P. lundensis* (19.8%), and *P. fluorescens* biovar III (17.3%) were the most commonly isolated species, while Marchand *et al.* (2009a) demonstrated that *P. lundensis* and *P. fragi* were the predominant milk spoilers in Belgian raw milk samples.

Similarly, He *et al.* (2009) found that pseudomonads predominated in cold stored pasteurised milk at 10 and 5 days before expiration as well as on the expiration day, although they also detected significant numbers of *Streptococcus* spp. and *Buttiauxella* spp. in all samples. Pseudomonads also predominated in the microbiota cultured from the crevices of cleaned devices sampled at a milk processing plant, demonstrating their potential roles as post-collection contaminants (Cleto *et al.*, 2012). The ability of this group of microbes to resist cleaning is linked to the fact that many species are effective biofilm producers (Bai and Rai, 2011; Simões *et al.*, 2008). The complex and multi-layered structures of biofilms allow the bacterial communities to live in a sessile and protected environment. Yet, when population densities in the biofilms become high, bacteria are released into the environment, providing a continuous source of planktonic bacteria capable of replication within milk (Bai and Rai, 2011).

## *Pseudomonas* spp.: Misidentification

A study of raw milk samples obtained along the cold chain of milk transportation (from farm, trucks, and silos) in Finland demonstrated that the majority (88%) of the isolated bacteria were psychrotrophic (Munsch-Alatossava and Alatossava, 2006). The authors employed two commercial phenotypic identification systems (API-20NE and BIOLOG GN2) and reported difficulties in obtaining confident identification of many of the isolates. This was particularly the case for fluorescent pigment-producing pseudomonads, where the biochemical results were considered to be doubtful. In view of this controversy, the authors recommended the use of genotypic identification systems in future studies.

A comparative evaluation of phenotypic and genotypic methods for the identification of 102 food-associated psychrotrophic *Pseudomonas* spp. clearly demonstrated that molecular methods provided superior results (Franzetti and Scarpellini, 2007). In this study, phenotypic data identified the bacteria as *P. fluorescens* or *P. putida,* with a single strain identified as *P. aeruginosa*. In contrast, sequencing of 16S rDNA in combination with restriction fragment length polymorphism (RFLP) analyses resulted in the identification of the bacteria as *P. jessenii*, *P. orientalis*, *P. migulae* and *P. chicorii,* and also confirmed the phenotypic data for the *P. aeruginosa* isolate.

The study of Hantsis-Zacharov and Halpern (2007) evaluated 264 bacteria collected from raw milk during different seasons. They reported the presence of representatives of seven classes of bacteria (*Gammaproteobacteria*, *Bacilli*, *Actinobacteria*, *Alphaproteobacteria*, *Betaproteobacteria*, *Flavobacteria* and *Sphingobacteria*), with 20% of the isolates considered to be novel species. *Pseudomonas* and *Acinetobacter* were recorded as the predominant genera among the Gram-negatives, with 33 and 29 isolates, respectively. Sequencing of the gene encoding 16S rRNA identified the majority (15) of the isolates as *P. putida*, and demonstrated the presence of the novel milk-associated species *P. sinxantha*, *P. brenneri* and *P. veronii*.

Marchand *et al.* (2009a) employed a polyphasic approach including molecular methods for the identification of the predominant producers of heat-resistant proteases in raw milk in Belgium. *P. fragi* and *P. lundensis* represented 53% of the producers of heat-resistant proteases, with *P. fluorescens* representing a minority of the isolates. The authors recommended the increased application of genotypic identification methods to ensure accuracy, and they also called for a revision of the taxonomic status of *P. fluorescens*. Furthermore, they considered it likely that misidentification of many proteolytic isolates as *P. fluorescens* in earlier studies using phenotypic characterisation had led to an overestimation of the importance of this species as a milk spoiler.

A further example of the difficulties associated with the identification of milk spoiling *Pseudomonas* species was provided by the study of Corrêa *et al.* (2011). Using phenotypic methods, the authors identified the highly proteolytic *Pseudomonas* strain 1A4R as either *P. asplenii* or *P. jenssenii*. The subsequent application of 16S rDNA sequencing revealed a level of 99% nucleotide sequence homology with *P. koreensis*, which forms part of the so-called *P. jenssenii* group. In view of the divergent data, strain 1A4R was classified as a *Pseudomonas* sp. belonging to the *P. jenssenii* group.

Improvements in identification at the species and sub-species levels should become possible through the use of highly specific typing methods such as the Multilocus Sequence Typing (MLST) scheme developed by Andreani *et al.* (2014) for the characterisation of the *P. fluorescens* group. However, it should be noted that the elevated costs of such sequencing-based methods is likely to limit their use, at least in the short term, to academic studies.

## Use of Molecular Tools to Elucidate the Ecology of Psychrotrophic Bacteria

Indigenous bacterial communities in raw milk, including potentially lipolytic and proteolytic psychrotrophs, are already present when the milk arrives at the dairy plant, making the rapid and accurate identification of the raw milk microbiota a necessary prerequisite for the elaboration of methods to circumvent spoilage (Van Der Vossen and Hofstra, 1996). Based on the observation that initial psychrotrophic counts of milk are frequently very low (Mcphee and Griffiths, 2011), more sensitive and efficient methods to evaluate the bacterial quality of raw milk are required to identify the causes of reduced shelf life and the deterioration of technological properties of milk during storage (Quigley *et al.*, 2013).

Traditional microbiological approaches to the study of psychrotrophs in milk based on phenotypic characterisation are time consuming, lack discriminatory power and sensitivity and are often ineffective in establishing a causal relationship between the contamination of the finished product and the environmental source (Dogan and Boor, 2003; Rasolofo *et al.*, 2010). Furthermore, the inability to discriminate between closely related organisms can lead to misidentification, and the slow turnaround time of the results makes phenotypic testing useful mainly for retrospective evaluation (Ercolini, 2004; Raats *et al.*, 2011).

Molecular analyses of microbes offer some advantages over phenotypic methods, including speed and the ability to provide precise identification of microorganisms from the genus to the strain level, depending on the system used. The discrimination between subspecies and strains is helpful for investigating the routes and sources of contamination (Ercolini, 2004; Rasolofo *et al.*, 2010). Molecular methods can be culture-dependent (nucleic acids are recovered from cultured microbes) or culture-independent (total bacterial DNA/RNA is extracted directly from an environmental sample, thereby providing information for the components of the microbiota that are unable to grow under laboratory conditions) (Ercolini, 2004; Ercolini, 2013; Quigley *et al.*, 2013; Raats *et al.*, 2011).

The concept of viable but non-culturable (VBNC) bacteria refers to bacteria with metabolic activity and the ability to reproduce under suitable conditions, but which lack the capability to produce visible growth under standard conditions. Interest in the role of VBNC in food spoilage has increased due to the observation that some disinfection procedures such as pasteurisation of milk and chlorination of water can cause bacteria to switch to the VBNC form (Ozcakir, 2007).

High-throughput sequencing of 16S rDNA and real-time quantitative PCR (qPCR) analysis were used in combination with flow cytometry to examine the microbial content of raw and pasteurised cow milk (Quigley *et al.*, 2013). In contrast to findings from culture-based studies (Ranieri *et al.*, 2009), the use of culture-independent methods demonstrated that the pasteurisation process resulted in an overall reduction in the number of pseudomonads rather than their complete elimination. Interestingly, the presence of pseudomonads in pasteurised milk has traditionally been considered to be the result of post-pasteurisation contamination (Van Tassell *et al.*, 2012). However, it is worth considering that the survival and subsequent transition from VBNC to fully viable cells may explain, at least in part, the frequent isolation of this genus from pasteurised milk.

Quigley *et al.* (2011) recently reviewed several culture-dependent and -independent methods applicable to milk and cheese. Among the culture-independent methods, denaturing gradient gel electrophoresis (DGGE) based on the separation of complex mixtures of PCR amplicons of the same size but with different nucleotide sequences has emerged as the most commonly used fingerprinting technique applied to the study of populations of psychrotrophic bacteria associated with milk and dairy products (Ercolini, 2004; Raats *et al.*, 2011; Rasolofo *et al.*, 2010). This technology provides a convenient means to obtain a comprehensive overview of the microbial populations in milk during cold storage, has confirmed the predominance of *Pseudomonas* spp. in pasteurised milk samples during their shelf life and has revealed a greater level of species diversity among the pseudomonads than had been previously indicated by culture-dependent methods (He *et al.*, 2009; Raats *et al.*, 2011). Furthermore, the application of DGGE to raw milk samples from three farm bulk tanks and three dairy plant silo tanks in Israel clearly demonstrated that refrigeration served to reduce the microbial diversity of raw milk, and showed that the predominance of *Pseudomonas* species was actually the end result of complex microbial successions that varied depending on the source of the milk (Raats *et al.*, 2011). In the case of farm samples, Gram-positive bacteria represented by the classes *Actinobacteria* and *Bacilli* predominated at the time of collection and were gradually overgrown by *Pseudomonas* species. In contrast, dairy plant tank samples were initially dominated by a variety of Gram-negative bacteria belonging to the *Gammaproteobacteria* class, with *Pseudomonas* and *Acinetobacter* species predominating within 48 h of refrigeration (Raats *et al.*, 2011). The predominance of psychrotolerant members of the class *Actinobacter* in the farm samples and their presence at lower levels in the silo samples was in agreement with data from DGGE-based examinations of raw milk samples in Canada (Rasolofo *et al.*, 2010) and of raw milk and fresh curd used for Fontina cheese production in Italy (Giannino *et al.*, 2009). Interestingly, cultivable and milk-associated members of this class of bacteria were previously reported to possess the ability to secrete heat-stable proteolytic and lipolytic enzymes (Hantsis-Zacharov and Halpern, 2007). Hence, it is possible that these bacteria may play a role in the spoilage processes during the early stages post-milk collection or may even be involved in the degradation of proteins and fats present in processed dairy products.

An alternative molecular fingerprinting method called random amplified polymorphic DNA-polymerase chain reaction (RAPD-PCR) was applied to 66 bacterial isolates from cold stored raw milk prior to nucleotide sequence analysis of the gene encoding the 16S ribosomal RNA (Ercolini *et al.*, 2009). In agreement with the results obtained with DGGE, this approach identified *Pseudomonas* spp. as the most common contaminant, but also identified *Hafnia alvei*, *Serratia marcersens*, *Citrobacter freundii*, *Staphylococcus* and *Lactococcus*.

## Conclusion

The role of pseudomonads and primarily *P. fluorescens* as the predominant psychrotrophs associated with the spoilage of cold stored milk and milk-derived products has been established through a long history of culture-based studies. However, these types of studies are often limited to the phenotypic characterisation of the most abundant isolates following their isolation as pure cultures. The validity of this approach has been questioned by recent findings using nucleotide sequence-based identification methods, which have shown that the diversity of *Pseudomonas* species involved in milk spoilage is much wider than was previously thought. Moreover, real time molecular analysis of microbial communities has shown that psychrotrophic species other than *Pseudomonas* predominate in milk during the early stages post-collection, and that the activities of those communities may play a role in the subsequent spoilage of milk.

The emergence of molecular methods has provided a new means with which to obtain an accurate global view of the microbial communities in milk. The application of these methods has revealed potential roles for genera other than *Pseudomonas* as important agents of milk spoilage at refrigeration temperatures. Moreover, it is likely that the mixed bacterial populations that are often present in the form of biofilms collaborate in the spoilage process via mechanisms based on quorum sensing. The practical implications for these essentially preliminary findings have yet to be elucidated. However, it is clear that the continued study of milk-associated psychrotrophs is required and should be encouraged in order to enhance and improve existing control methods and to help ensure the quality and safety of milk and milk-derived foodstuffs.
